# Supplementary material for: The effectiveness of pharmacological and non-pharmacological interventions for fatigue in people living with chronic kidney disease: A protocol for a systematic review
Source: PLoS One. 2026 May 5;21(5):e0348585. doi: 10.1371/journal.pone.0348585 (PMC13143074; doi:10.1371/journal.pone.0348585)
Supplement: S2 Table — (DOCX) [file pone.0348585.s002.docx]

# Supplementary Material 2: Draft Medline search strategy

1 Renal Insufficiency, Chronic/

2 Kidney Failure, Chronic/

3 (Kidney adj2 (end-stage or end stage or chronic or conservative)).ti,ab,kf.

4 (Renal adj2 (end-stage or end stage or chronic or conservative)).ti,ab,kf.

5 (CKD or CRF or ESKD or ESRF).ti,ab,kf.

6 Renal Replacement Therapy/

7 exp Renal Dialysis/

8 Hemodiafiltration/

9 Hemodialysis, home/

10 dialysis.ti,ab,kf.

11 Renal replacement therap*.ti,ab,kf.

12 Kidney replacement therap*.ti,ab,kf.

13 (hemodialysis or haemodialysis).ti,ab,kf.

14 (hemofiltration or haemofiltration).ti,ab,kf.

15 (hemodiafiltration or haemodiafiltration).ti,ab,kf.

16 exp Peritoneal Dialysis/

17 (CAPD or APD or CCPD).ti,ab,kf.

18 Kidney Transplantation/

19 Kidney transplant*.ti,ab,kf.

20 Renal transplant*.ti,ab,kf.

21 ((without or refus*) adj2 dialys*).ab,kf,ti.

22 1 or 2 or 3 or 4 or 5 or 6 or 7 or 8 or 9 or 10 or 11 or 12 or 13 or 14 or 15 or 16 or 17 or 18 or 19 or 20 or 21

23 fatigue/ or emotional exhaustion/ or mental fatigue/

24 (fatigue$ or astheni$ or neurastheni$ or tired or tiredness or tire or weary or weariness or exhausted or exhaustion or lassitude or listlessness or letharg$ or apath$ or malaise or lacklustre or lackluster or weak or weakness).ti,ab,kf.

25 ((loss or low or lack*) adj2 (vitality or energy or vigor* or vigour*)).ti,ab,kf.

26 (feel* adj3 (drained or sluggish*)).ti,ab,kf.

27 23 or 24 or 25 or 26

28 22 and 27

29 (Randomized Controlled Trial or Controlled Clinical Trial or Pragmatic Clinical Trial or Clinical Study or Adaptive Clinical Trial or Equivalence Trial).pt.

30 (Clinical Trial or Clinical Trial, Phase I or Clinical Trial, Phase II or Clinical Trial, Phase III or Clinical Trial, Phase IV or Clinical Trial Protocol).pt.

31 Multicenter Study.pt.

32 Clinical Studies as Topic/

33 exp Clinical Trial/ or exp Clinical Trials as Topic/ or Clinical Trial Protocol/ or Clinical Trial Protocols as Topic/ or exp "Clinical Trial (topic)"/

34 Multicenter Study/ or Multicenter Studies as Topic/ or "Multicenter Study (topic)"/

35 Randomization/

36 Random Allocation/

37 Double-Blind Method/

38 Double Blind Procedure/

39 Double-Blind Studies/

40 Single-Blind Method/

41 Single Blind Procedure/

42 Single-Blind Studies/

43 Placebos/

44 Placebo/

45 Control Groups/

46 Control Group/

47 Cross-Over Studies/ or Crossover Procedure/

48 (random* or sham or placebo*).ti,ab,hw,kf.

49 ((singl* or doubl*) adj (blind* or dumm* or mask*)).ti,ab,hw,kf.

50 ((tripl* or trebl*) adj (blind* or dumm* or mask*)).ti,ab,hw,kf.

51 (control* adj3 (study or studies or trial* or group*)).ti,ab,hw,kf.

52 (clinical adj3 (study or studies or trial*)).ti,ab,hw,kf.

53 (Nonrandom* or non random* or non-random* or quasi-random* or quasirandom*).ti,ab,hw,kf.

54 (phase adj6 (study or studies or trial*)).ti,ab,hw,kf.

55 ((crossover or cross-over) adj3 (study or studies or trial*)).ti,ab,hw,kf.

56 ((multicent* or multi-cent*) adj3 (study or studies or trial*)).ti,ab,hw,kf.

57 allocated.ti,ab,hw.

58 ((open label or open-label) adj5 (study or studies or trial*)).ti,ab,hw,kf.

59 ((equivalence or superiority or non-inferiority or noninferiority) adj3 (study or studies or trial*)).ti,ab,hw,kf.

60 (pragmatic study or pragmatic studies).ti,ab,hw,kf.

61 ((pragmatic or practical) adj3 trial*).ti,ab,hw,kf.

62 ((quasiexperimental or quasi-experimental) adj3 (study or studies or trial*)).ti,ab,hw,kf.

63 trial.ti,kf.

64 29 or 30 or 31 or 32 or 33 or 34 or 35 or 36 or 37 or 38 or 39 or 40 or 41 or 42 or 43 or 44 or 45 or 46 or 47 or 48 or 49 or 50 or 51 or 52 or 53 or 54 or 55 or 56 or 57 or 58 or 59 or 60 or 61 or 62 or 63

65 28 and 64

66 exp Empirical Research/ or Interviews as Topic/ or Personal Narratives as Topic/ or Focus Groups/ or exp Narration/ or Nursing Methodology Research/ or Narrative Medicine/

67 (Interview or Personal Narrative).pt.

68 interview*.ti,ab,kf.

69 qualitative.ti,ab,kf,jw.

70 (theme* or thematic).ti,ab,kf.

71 ethnological research.ti,ab,kf.

72 ethnograph*.ti,ab,kf.

73 ethnomedicine.ti,ab,kf.

74 ethnonursing.ti,ab,kf.

75 phenomenol*.ti,ab,kf.

76 (grounded adj (theor* or study or studies or research or analys?s)).ti,ab,kf.

77 life stor*.ti,ab,kf.

78 (emic or etic or hermeneutic* or heuristic* or semiotic*).ti,ab,kf.

79 (data adj1 saturat$).ti,ab,kf.

80 participant observ*.ti,ab,kf.

81 (social construct* or postmodern* or post-structural* or post structural* or poststructural* or post modern* or post-modern*).ti,ab,kf.

82 (action research or cooperative inquir* or co operative inquir* or co-operative inquir*).ti,ab,kf.

83 (humanistic or existential or experiential or paradigm*).ti,ab,kf.

84 (field adj (study or studies or research or work)).ti,ab,kf.

85 (human science or social science).ti,ab,kf.

86 biographical method.ti,ab,kf.

87 theoretical sampl*.ti,ab,kf.

88 ((purpos* adj4 sampl*) or (focus adj group*)).ti,ab,kf.

89 (open-ended or narrative* or textual or texts or semi-structured).ti,ab,kf.

90 (life world* or life-world* or conversation analys?s or personal experience* or theoretical saturation).ti,ab,kf.

91 ((lived or life) adj experience*).ti,ab,kf.

92 cluster sampl*.ti,ab,kf.

93 observational method*.ti,ab,kf.

94 content analysis.ti,ab,kf.

95 (constant adj (comparative or comparison)).ti,ab,kf.

96 ((discourse* or discurs*) adj3 analys?s).ti,ab,kf.

97 (heidegger* or colaizzi* or spiegelberg* or merleau* or husserl* or foucault* or ricoeur or glaser*).ti,ab,kf.

98 (van adj manen*).ti,ab,kf.

99 (van adj kaam*).ti,ab,kf.

100 (corbin* adj2 strauss*).ti,ab,kf.

101 66 or 67 or 68 or 69 or 70 or 71 or 72 or 73 or 74 or 75 or 76 or 77 or 78 or 79 or 80 or 81 or 82 or 83 or 84 or 85 or 86 or 87 or 88 or 89 or 90 or 91 or 92 or 93 or 94 or 95 or 96 or 97 or 98 or 99 or 100

102 28 and 101

103 65 or 102

104 Epidemiologic Methods/

105 exp Epidemiologic Studies/

106 Observational Studies as Topic/

107 Clinical Studies as Topic/

108 single-case studies as topic/

109 case reports as topic/

110 (Observational Study or Validation Studies or Clinical Study).pt.

111 (observational adj3 (study or studies or design or analysis or analyses)).ti,ab,kf.

112 cohort*.ti,ab,kf.

113 (prospective adj7 (study or studies or design or analysis or analyses)).ti,ab,kf.

114 ((follow up or followup) adj7 (study or studies or design or analysis or analyses)).ti,ab,kf.

115 ((longitudinal or longterm or (long adj term)) adj7 (study or studies or design or analysis or analyses or data)).ti,ab,kf.

116 (retrospective adj7 (study or studies or design or analysis or analyses or data or review)).ti,ab,kf.

117 ((case adj control) or (case adj comparison) or (case adj controlled)).ti,ab,kf.

118 (case-referent adj3 (study or studies or design or analysis or analyses)).ti,ab,kf.

119 (population adj3 (study or studies or analysis or analyses)).ti,ab,kf.

120 (descriptive adj3 (study or studies or design or analysis or analyses)).ti,ab,kf.

121 ((multidimensional or (multi adj dimensional)) adj3 (study or studies or design or analysis or analyses)).ti,ab,kf.

122 (cross adj sectional adj7 (study or studies or design or research or analysis or analyses or survey or findings)).ti,ab,kf.

123 ((natural adj experiment) or (natural adj experiments)).ti,ab,kf.

124 (quasi adj (experiment or experiments or experimental)).ti,ab,kf.

125 ((non experiment or nonexperiment or non experimental or nonexperimental) adj3 (study or studies or design or analysis or analyses)).ti,ab,kf.

126 (prevalence adj3 (study or studies or analysis or analyses)).ti,ab,kf.

127 case series.ti,ab,kf.

128 case reports.pt.

129 (case adj3 (report or reports or study or studies or histories)).ti,ab,kf.

130 organizational case studies/

131 104 or 105 or 106 or 107 or 108 or 109 or 110 or 111 or 112 or 113 or 114 or 115 or 116 or 117 or 118 or 119 or 120 or 121 or 122 or 123 or 124 or 125 or 126 or 127 or 128 or 129 or 130

132 28 and 131

133 103 or 132
