# Supplementary material for: The effectiveness of pharmacological and non-pharmacological interventions for fatigue in people living with chronic kidney disease: A protocol for a systematic review
Source: PLoS One. 2026 May 5;21(5):e0348585. doi: 10.1371/journal.pone.0348585 (PMC13143074; doi:10.1371/journal.pone.0348585)
Supplement: Attachment — Submitted filename: fatigue protocol responses to reviewers final.docx [file pone.0348585.s003.docx]

| **Journal requirements:** | | |
| --- | --- | --- |
| Comment raised ^i ^ | Response by author ^i ^ | Location of revisions ^i^ |
| 1. When submitting your revision, we need you to address these additional requirements.    Please ensure that your manuscript meets PLOS ONE's style requirements, including those for file naming. The PLOS ONE style templates can be found at  https://journals.plos.org/plosone/s/file?id=wjVg/PLOSOne_formatting_sample_main_body.pdf and  https://journals.plos.org/plosone/s/file?id=ba62/PLOSOne_formatting_sample_title_authors_affiliations.pdf | Thank you for highlighting this. We have been through the manuscript to ensure it complies with the style requirements for Plos One, including file names. | Throughout manuscript |
| 2. Please provide a complete Data Availability Statement in the submission form, ensuring you include all necessary access information or a reason for why you are unable to make your data freely accessible. If your research concerns only data provided within your submission, please write "All data are in the manuscript and/or supporting information files" OR If no pilot data are reported in the manuscript, “No datasets were generated or analysed during the current study. All relevant data from this study will be made available upon study completion” as your Data Availability Statement. | Thank you – we have included the following data availability statement in the submission form: The current manuscript is a study protocol for a systematic review and meta-analysis which is still currently in progress. No results are available to be reported yet at current stage. All deidentified research data will be made publicly available when the study is completed and published | Submission form |
| 3. Please include captions for your Supporting Information files at the end of your manuscript, and update any in-text citations to match accordingly. Please see our Supporting Information guidelines for more information: <http://journals.plos.org/plosone/s/supporting-information>. | We have now completed this as per the formatting guidance | Page 21 |
| **Reviewer 1** | | |
| The scope of this project is very broad and heterogeneous. The authors plan to assess all interventions targeting fatigue across all stages of CKD (eGFR < 60 mL/min/1.73 m²), including randomized controlled trials, non-randomized trials, and prospective studies, and additionally to conduct a qualitative systematic review of patient perspectives. While this ambition is commendable, the research question may be too wide and complex to address adequately within a single SRMA. A more focused scope—or clearer justification for combining such diverse interventions, populations, and study designs—would strengthen the protocol. | We acknowledge that the protocol is broad, however the reason for this is that it will ultimately be used to inform the development of the UK Kidney Association guideline, which is targeted at a broad audience, including patients and clinicians who are treating people with different CKD etiology. We have now clarified this intent.  To our best knowledge, the literature available on effectiveness of different interventions which may impact fatigue for each pre-defined CKD subpopulation is scarce, and hence we have chosen to include a range of study designs. | Page 5, line 18. |
| The introduction is overly long and would benefit from substantial condensation. It should be shortened to approximately one to one and a half pages, focusing more concisely on the rationale, existing gaps in the literature, and the specific objectives of the review. | Thank you for your feedback we have now shortened the protocol, with more of a focus on the rationale, retaining information about or intention to inform a clinical guideline (in light of your first point), gaps in the literature and the objectives of the review. | Pages 3-6 |
| The authors did not provide a sufficiently detailed description of the keywords and search terms that will be used. Including the full search strategy for at least one database would improve transparency and reproducibility. | We include the full search strategy for Medline within supplementary material two and this is outlined on page 9, line 27. We would be happy to include other information if you could clarify what is required. | No changes |
| The protocol was registered on PROSPERO in September 2025, yet the authors state that results will be available in July 2025. This appears to be a chronological error and should be clarified. | Thank you for spotting this and apologies this is a typo we have now corrected | Page 17, lines 445 |
| **Reviewer 2** | | |
| Multiple patient eligibility criteria (all CKD stages, HD, PD, transplanted patients…) and both pharmacological and non-pharmacological interventions are eligible, across multiple study designs (RCTs, non-randomised studies, before-after studies, qualitative studies). The feasibility of synthesising such diverse populations and interventions is uncertain. | Thank you for your comments. We hope that in our response to reviewer one on their similar comment we have now sufficiently addressed these concerns | See previous changes related to reviewer one’s comments |
| Provide a clearer justification of the transitivity assumption underpinning the proposed NMA. | Thank you for this comment – we have now justified this also follows: *“The validity of indirect comparisons relies on the transitivity assumption. Although variations in study populations (CKD stage) and intervention characteristics are anticipated, the included treatments will share similar therapeutic objectives. Therefore, it is considered plausible that relative treatment effects remain comparable across studies.*  *Important clinical and methodological characteristics that may be effect modifiers (including but not limited to demographics, CKD stage, intervention intensity, and study design) will be examined across comparisons. Where substantial heterogeneity is identified, subgroup analyses or meta-regression will be conducted where feasible. If, after these explorations, unexplained heterogeneity remains that undermines the assumptions of consistency and transitivity, the NMA results will not be reported. Transitivity will be assessed by comparing the distribution of effect modifiers across treatment comparisons. In cases of disconnected networks, analyses will focus on the largest connected network or be conducted separately for each network. Results will be interpreted with appropriate caution”* | Page 14, lines 348-360 |
| Clarify whether analyses will be stratified a priori by CKD population (e.g., dialysis vs non-dialysis vs transplant). | Thank you for raising this important point – we have now clarified this as follows for the primary outcome:  *“In view of the heterogeneity of fatigue syndrome across chronic kidney disease cohorts, the primary outcome will be reported separately for four groups: patients undergoing dialysis, non-dialysis patients (including those in pre-dialysis stages), kidney transplant recipients, and individuals with CKD receiving conservative management and for adults and children. The primary outcome will likewise be analysed separately according to categories of pharmacological and non-pharmacological interventions.”*  And for the secondary outcomes:  *“All secondary analyses will also be stratified by CKD stage, as per the primary outcome, and by age group (adult/child).”* | Page 8, lines 197-202 and page 9 lines 222-223 |
| Define conditions under which NMA would be deemed inappropriate due to heterogeneity. | Thank you for your comment – we have now clarified this:  *‘If, after these explorations, unexplained heterogeneity remains that undermines the assumptions of consistency and transitivity, the NMA results will not be reported.’* | Page 14, lines 355-357 |
| The protocol adopts a broad definition of fatigue. While this enhances inclusivity, it raises conceptual and statistical concerns: fatigue-specific instruments are not equivalent to fatigue subscales and different instruments may measure distinct fatigue dimensions. The authors should: - Define a hierarchy of outcome measures (e.g., fatigue-specific validated instruments as primary). - Pre-specify how multidimensional fatigue instruments will be handled. - Consider sensitivity analyses by instrument type. | We thank the reviewer for this insightful comment. We agree that the use of a broad definition of fatigue may introduce conceptual and methodological heterogeneity. To address this concern, we have strengthened the protocol in three ways:   1. We now predefine a hierarchy of fatigue outcomes. Fatigue-specific validated instruments are designated as primary outcomes, while fatigue subscales derived from multidimensional instruments and single-item measures are treated as secondary outcomes. 2. We have clarified that when multidimensional instruments are used, only validated fatigue-specific subscales will be extracted. When appropriate (and if possible), domains will be analysed separately to avoid conceptual conflation. 3. We will conduct sensitivity analyses stratified by instrument category (fatigue-specific vs multidimensional subscales vs single-item measures) to examine the robustness of pooled estimates and assess potential measurement-related heterogeneity. | Page 8, lines 189-194, page 16 lines 397-400 |
| The protocol should clarify: - Whether a frequentist or Bayesian NMA framework will be used. - How multi-arm trials will be handled. - How combination interventions will be classified. | Thank you for your comment. We have now specified that:   - ‘*Where appropriate, data from randomised controlled trials using comparable outcomes will be pooled in meta-analyses within a Frequentist framework’*. - That *‘Multi-arm studies will be accounted for by reweighting all comparisons of each multi-arm study.’* - *‘Interventions that combine multiple approaches will be classified as distinct interventions within the analysis.’* | Page 13, lines 317-318.  Page 14, lines 339-340  Page 14, lines 338-340 |
| The protocol states that « Searches were conducted in July 2025. Screening anticipated completion in January 2025. Results expected July 2025. This timeline is internally inconsistent and must be corrected. | Thank you for your comments. We hope that in our response to reviewer one on their similar comment we have now sufficiently addressed these concerns | See previous changes related to reviewer one’s comments |
